# RNA Methylations in Cardiovascular Diseases, Molecular Structure, Biological Functions and Regulatory Roles in Cardiovascular Diseases

**DOI:** 10.3389/fphar.2021.722728

**Published:** 2021-08-19

**Authors:** Wanwan Zhou, Changhui Wang, Jun Chang, Yurong Huang, Qiuyun Xue, Chenggui Miao, Peng Wu

**Affiliations:** ^1^ Department of Pharmacology, School of Integrated Chinese and Western Medicine, Anhui University of Chinese Medicine, Hefei, China; ^2^ Department of Cardiology, The First Affiliated Hospital, Anhui Medical University, Hefei, China; ^3^ Department of Orthopaedics, The Fourth Affiliated Hospital, Anhui Medical University, Hefei, China; ^4^ Anhui Provincial Key Laboratory of Applied Basis and Development of Modern Internal Medicine of Traditional Chinese Medicine, The First Affiliated Hospital, Anhui University of Chinese Medicine, Hefei, China; ^5^ Department of Anatomy, School of Integrated Chinese and Western Medicine, Anhui University of Chinese Medicine, Hefei, China

**Keywords:** cardiovascular disease, RNA methylation, m6A, m5C, pulmonary hypertension

## Abstract

Cardiovascular diseases (CVDs) are the leading cause of morbidity and mortality in the world. Despite considerable progress in the diagnosis, treatment and prognosis of CVDs, new diagnostic biomarkers and new therapeutic measures are urgently needed to reduce the mortality of CVDs and improve the therapeutic effect. RNA methylations regulate almost all aspects of RNA processing, such as RNA nuclear export, translation, splicing and non-coding RNA processing. In view of the importance of RNA methylations in the pathogenesis of diseases, this work reviews the molecular structures, biological functions of five kinds of RNA methylations (m6A, m5C, m1a, m6am and m7G) and their effects on CVDs, including pulmonary hypertension, hypertension, vascular calcification, cardiac hypertrophy, heart failure. In CVDs, m6A “writers” catalyze the installation of m6A on RNAs, while “erasers” remove these modifications. Finally, the “readers” of m6A further influence the mRNA splicing, nuclear export, translation and degradation. M5C, m1A, m6Am and m7G are new types of RNA methylations, their roles in CVDs need to be further explored. RNA methylations have become a new research hotspot and the roles in CVDs is gradually emerging, the review of the molecular characteristics, biological functions and effects of RNA methylation on CVDs will contribute to the elucidation of the pathological mechanisms of CVDs and the discovery of new diagnostic markers and therapeutic targets of CVDs.

## Introduction

Cardiovascular diseases (CVDs) are the leading cause of morbidity and mortality in the world, and the pathological mechanisms are complicated (Noale et al., 2020). CVDs represent a series of diseases of circulatory system, including pulmonary hypertension, hypertension, vascular calcification, cardiac hypertrophy, cardiac arrhythmias, atherosclerosis, angina pectoris, myocardial infarction, heart failure (Senoner and Dichtl, 2019; Xu et al., 2019; Du et al., 2020). There are a group of clear risk factors in the pathogenesis of CVDs, such as high glucose level, elevated blood pressure, dyslipidemia, overweight and persistent inflammation of diseased tissues (Altesha et al., 2019).

Pulmonary hypertension (PH) refers to a hemodynamic and pathological state in which the pulmonary artery pressure rises beyond the threshold, which can lead to right heart failure. The hemodynamic diagnostic criteria of PH is mean pulmonary artery pressure ≥25 mmHg (Mandras et al., 2020). PH can be an independent disease or a complication, and the PH is a disease with high morbidity and mortality. PH are nonspecific and asymptomatic in the early stage. As the disease progresses, the disease symptoms include dyspnea, fatigue, decreased exercise tolerance, syncope, angina pectoris, chest pain and right heart failure (Cassady and Ramani, 2020).

Hypertension is a clinical syndrome characterized by increased systemic arterial blood pressure (systolic blood pressure ≥140 mmHg, diastolic blood pressure ≥90 mmHg), which can be accompanied by damage of heart, brain, kidney and other organs (Lamirault et al., 2020; Song et al., 2020). Hypertension is the most common chronic disease and the main risk factor of CVDs. The blood pressure of hypertension patients fluctuates in a certain range with the changes of internal and external environment. Hypertension may be asymptomatic or not obvious in the early stage, but dizziness, headache, fatigue and palpitation can be seen in later stage. Hypertension can occur after fatigue, mental tension and emotional fluctuation, and it will return to normal after rest (Agrawal and Wenger, 2020).

Vascular calcification is a common pathological manifestation of atherosclerosis, hypertension, diabetic vascular disease, vascular injury, chronic kidney disease and aging. The main symptoms of vascular calcification are increased vascular wall sclerosis and decreased compliance, which easily lead to myocardial ischemia, left ventricular hypertrophy and heart failure (Lee et al., 2020). Thrombosis and plaque rupture caused by vascular calcification are important factors of high incidence rate and high mortality rate of CVDs. Vascular calcification is also an important marker of atherosclerotic cardiovascular events, stroke and peripheral vascular disease (Zununi Vahed et al., 2020).

Cardiac hypertrophy mainly occurs in the case of long-term myocardial pressure overload, the total amount of myocardium increases and the contractility strengthens. Cardiac hypertrophy enables the heart to maintain normal blood circulation. Cardiac hypertrophy leads to the increase of myocardial oxygen demand, and the blood supply of coronary artery is often unable to supply, resulting in myocardial ischemia. This eventually lead to a decrease in myocardial contractility (Wu et al., 2020a). The main symptoms of cardiac hypertrophy include dyspnea, chest pain, fatigue, dizziness and fainting, palpitation and heart failure. The disease characteristics of advanced patients are extensive myocardial fibrosis and weakened ventricular systolic function, leading to heart failure and sudden death (Matsuura et al., 2020).

Heart failure refers to the failure of cardiac systolic function and (or) diastolic function to fully discharge venous blood from the heart, eventually leads to venous system blood stasis, insufficient arterial blood perfusion (Murphy et al., 2020). Heart failure further leads to cardiac circulation disorder, which is manifested as pulmonary congestion and venous congestion. Heart failure is not an independent disease, but the end stage of the development of a variety of heart diseases. The vast majority of heart failure begins with left heart failure (Sinnenberg and Givertz, 2020).

For patients with CVDs, it is recommended to take preventive measures to reduce the risk, such as lifestyle changes. On the other hand, traditional drugs such as renin angiotensin blockers, lipid-lowering drugs, beta receptor inhibitors and antithrombotic drugs are used to prevent disease progression (Blanda et al., 2020). For a long time, people have been trying to prevent the occurrence and development of CADs. However, none of these treatments has really solved the problem of CAD incidence rate rising. Basic scientific research is focusing on identifying the potential therapeutic targets of these diseases, and deeply and comprehensively understanding the pathological mechanisms is particularly urgent (Che et al., 2020; Jang et al., 2020).

RNA methylations regulate almost all aspects of RNA processing, including nuclear export, RNA translation, splicing and non-coding RNA processing (Lan et al., 2019). Because of the availability and understanding of new detection technologies, the relationship between RNA methylations and basic genetic processes is gradually revealed (Sun et al., 2019).

Methyl modifications of RNAs form post transcriptional regulatory mechanisms, which can fine tune gene expression by altering the interaction between RNAs and other components of cells. RNA methylations involve the “writers”, “erasers”, and “readers” (Michalak et al., 2019). Although the structures and functions of many RNA species depend on their methylations, RNA methylations seem to be dynamic to some extent, allowing fine-tuning of protein coding genes and cellular processes. These modifications do not affect the coding sequence, but affect the expression characteristics of transcripts, and widely affect the regulation of gene expression (Kmietczyk et al., 2019; Shi et al., 2019).

M1A, m6Am and m7G are new types of RNA methylations. M1A is a highly abundant post transcriptional modifications of tRNAs and rRNAs in eukaryotes. M1A modification affects the regulation of mRNA translation. M6Am is an evolutionarily conserved mRNA modification, which is different from m6A in function. M6Am does not alter mRNA transcription or stability, but negatively affects the cap dependent translation of methylated mRNAs (Tan and Gao, 2018). M7G methylation regulates the mRNA transcription, miRNA biosynthesis and biological functions, tRNA stability, 18S rRNA processing and maturation (Tomikawa, 2018). As new types of RNA methylations, the relationship between m1A, m6Am and m7G modifications and CVDs has not been reported, and their functions and mechanisms urgently need to be explored in future.

In view of the fact that RNA methylations have become new research hotspots and their functions has been emerged in CVDs are gradually emerging, this work focuses on the roles and mechanisms of RNA methylations (m6A, m5C, m1A, m6Am and m7G) in CVDs, and reveals the molecular characteristics, biological functions and the effects on these diseases.

## RNA Methylation

Methylation is an important modification of nucleic acids and proteins, which regulates gene expression and closure. It is closely related to many diseases, such as cancer, aging, Alzheimer’s disease (AD) (Greenberg and Bourc'his, 2019). Similar to the mechanisms of DNA methylations, RNA methylations refer to the alkylation form of adding methyl or substituting original atoms or groups on the substrate (Chi et al., 2018). In biological system, RNA methylations lead to the epigenetic changes to regulate the gene expression, but do not lead to changes in gene sequence (Ovcharenko and Rentmeister, 2018). Here, some well-known RNA methylations in mRNAs and molecular structures are introduced, including m6A, m5C, m1A, m6Am and m7G.

Furthermore, RNA methylations exist in many RNA species. For example, besides mRNA, m5C also exists in ribosomal RNAs (rRNAs), transfer RNAs (tRNAs) and some non-coding RNAs (Bohnsack et al., 2019; Wnuk et al., 2020). In addition to the m1A in mRNAs, this RNA modification is also crucial for the stability of tRNA. M1A occurs at positions 9, 14 and 58 of tRNA, and the m1A58 is involved in the stability of tRNA (Oerum et al., 2017; Zhang and Jia, 2018a).

### M6A Modification

M6A refers to the methylation of nitrogen-6 adenosine base (Cao et al., 2016; Dominissini et al., 2017). Up to now, m6A is considered to be the most abundant methylation modification in mRNAs. About 25% of mRNAs carry at least one m6A site. Both mRNAs and lncRNAs are heavily modified by m6A (Meyer et al., 2012). M6A is dynamically regulated by specific methyltransferases and demethylases, which cooperate with each other to maintain appropriate mRNA methylation status (Coker et al., 2019). The most characteristic methyltransferase that has been identified on mRNA is the methyltransferase complex composed of methyltransferase-like 3 (METTL3) and methyltransferase-like 14 (METTL14). METTL3 is a catalytic subunit, a s-adenosine-l-methionine (SAM) binding protein. METTL14 is a complex subunit that promotes the RNA binding (Zeng et al., 2020). The third important writer for mRNA is the Wilms tumor 1-associating protein (WTAP). WTAP interacts with METTL3-METTL14 and is also the third subunit of METTL3-METTL14 complex (Zhao et al., 2020).

**FIGURE 1:**
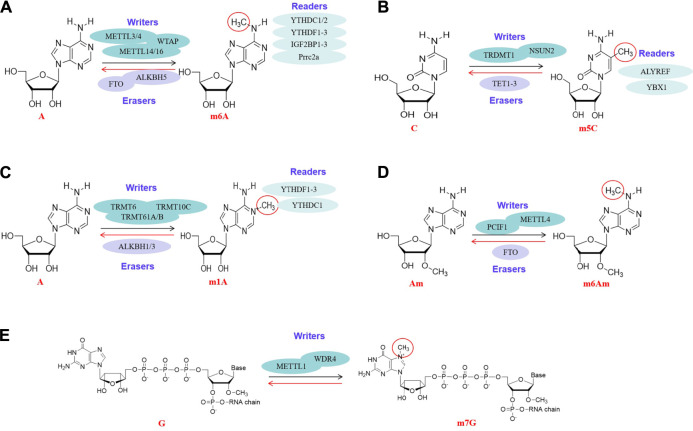
Molecular structures of RNA methylations. All kinds of RNA methylation modifications are mediated by enzymes, including methylases (“writers”), demethylases (“erasers”) and methylation recognition enzymes (“readers”). Methylases and demethylases coordinate the reversible and dynamic changes of RNA methylations, and methylation recognition enzymes are responsible for reading methylation sites and subsequent functions. The RNA methylations involved are m6A (Figure 2A), m5C (Figure 2B), m1A (Figure 2C), m6Am (Figure 2D), m7G (Figure 2E).

Fat mass and obesity-associated protein (FTO) is the first m6A eraser discovered in mRNAs (Jia et al., 2011). FTO catalyzes the oxidation of m6A to N(6)-hydroxymethyladenosine (hm6A), and further oxidizes the hm6a to N(6)-formyladenosine (Deng et al., 2018). Another eraser in mRNAs and tRNAs is the AlkB homolog 5 (ALKBH5), a well-known demethylase. ALKBH5 is characterized by direct demethylation from methylated adenosine rather than oxidative demethylation similar to FTO (Ueda et al., 2017; Wang et al., 2020a; Li et al., 2021a). (Figure 1A).

### M5C Modification

M5C exists in RNA by introducing a methyl group into the fifth carbon atom of cytosine (Dou et al., 2020). M5C is a rich RNA modification widely existing in many species, including cytoplasmic and mitochondrial rRNAs, tRNAs, mRNAs and non-coding RNAs. The m5C site in mRNAs is usually located near the Argonaute binding region in 3′ UTR or translation initiation site (Yang et al., 2020). Although the NSUN1, NSUN2 and NSUN5 of NSUN seven members (NSUN1-7) are conserved in eukaryotes, the remaining NSUNs only exist in higher eukaryotes. NSUN family is a SAM-dependent methyltransferase, which is represented by RNA recognition motif (RRM) and Rossman folding catalytic core containing SAM cofactors (Li et al., 2019a).

The m5C methyltransferases TRDMT1 is known to modify specific tRNAs and have roles in the control of cell growth and differentiation (Squires et al., 2012). Furthermore, 5-hydroxymethylcytosine (hm5C), a ten-eleven translocation (TET) mediated oxidation product, was specifically enriched in tRNA. TET2 mediated oxidation of m5C in tRNA promotes translation *in vitro*. This suggests that TET2 may affect translation by affecting tRNA methylation, and reveals the roles of TET enzyme in regulating gene expression (Shen et al., 2021a) (Figure 1B).

### M1A Modification

M1A changes the secondary structure of RNAs and affects the interaction between RNAs and proteins by adding a methyl group to the N1 position of adenosine. It’s a reversible methylation (Wei et al., 2018; Dong and Cui, 2020). Unlike m6A, m1A is found in rRNAs, tRNAs, mRNAs and mitochondrial RNAs. Interestingly, the content of m1A is higher in tRNAs and rRNAs, but lower in mRNAs. The ratio of m1A/A in mammalian cells is about 0.02%, and the highest is 0.16% (Shima and Igarashi, 2020). M1A is a post transcriptional modification with high abundance in tRNAs and rRNAs of eukaryotes. Recent studies have also shown that m1A modification can regulate the mRNA translation (Shi et al., 2020a).

The m1A methyltransferases found in cytoplasmic tRNAs are TRMT6/TRMT61A, TRMT61B, TRMT10C. TRMT6 plays an important role in tRNA binding. TRMT61A has the catalytic activity of tRNA adenine-N1-methyltransferase. TRMT61B and TRMT10C catalyze m1A at positions 9 and 58 of mitochondrial tRNAs. Furthermore, these tRNA methyltransferases can also catalyze m1A in mRNAs. For example, the TRMT6/61A add the m1A to mRNAs carrying the guucra tRNA like motif, while TRMT61B and TRMT10C write m1A to some mitochondrial mRNAs (Dégut et al., 2016). ALKBH1 and ALKBH3 may be the erasers of m1A, which can eliminate the modification of m1A, but the mechanisms are slightly different. ALKBH1 is a m1A demethylase in tRNAs, while the ALKBH3 demethylation targets mRNAs and tRNAs (Chen et al., 2019d). In addition, YTHDF3 inhibits trophoblast invasion by down-regulating the m1A methylated insulin-like growth factor 1 receptor (IGF1R) (Dai et al., 2018; Zheng et al., 2020) (Figure 1C).

### M6Am Modification

M6Am is found at the 5ʹend of up to 30% of mRNAs. The first nucleotide after m7G cap can be methylated on ribose to form 2′-O-methyladenosine (Am). Then, Am can be further methylated at N6 to generate m6Am (Mauer et al., 2017; Koh et al., 2019). Unlike m6A, m6Am is mainly located at the first base after the 5ʹ end cap of eukaryotic mRNAs. The ratios of m6Am/A of total RNAs in human tissue range from 0.0036 to 0.0169%, and the level of m6Am is negatively correlated with the corresponding protein expression. M6Am is a dynamic and reversible methylation modification, which has the same ability to affect gene expression as m6A (Akichika et al., 2019; Liu et al., 2020a). Deletion of methyltransferase METTL3 in adult neurons alters the transcription of m6Am RNAs (Engel et al., 2018). METTL4 is a snRNA m6Am methyltransferase, which catalyzes the methylation of m6Am in U2 snRNA to regulate pre-mRNA splicing (Chen et al., 2020; Goh et al., 2020).

FTO controls the reversible methylation of m6Am mRNAs during snRNA biogenesis. The biogenesis of snRNA begins with the formation of an initial M1 subtype of a single methylated adenosine (2′-O-methyladenosine, Am), and then the M1 subtype is transformed into a dimethyl M2 subtype (N6,2′-O-dimethyladenosine, m6Am). The relative levels of M1 and M2 subtypes were determined by FTO, which selectively demethylated M2 subtypes, suggesting that FTO plays an important role in the biogenesis of snRNA (Mauer et al., 2019). Obviously, PCIF1, METTL3 and METTL4 play the roles of writers of m6Am, and FTO is the eraser of m6Am in mRNA (Figure 1D).

### M7G Modification

Most eukaryotic mRNAs have a methyl group and a positive charge at the N7 position of the 5′ terminal guanine nucleoside. M7G RNA methylation is a kind of modification that makes the seventh N of RNA guanine adds methyl under the action of methyltransferases (Enroth et al., 2019). Studies have shown that the methylation of m7G exists in various kinds of molecules, including mRNA 5′cap structure, mRNAs, pri-miRNAs, tRNAs and rRNAs. M7G methylation can regulate mRNA transcription, miRNA biosynthesis and biological functions, tRNA stability, 18S rRNA processing and maturation (Chen et al., 2019c).

METTL1 mediated methylation enhances the let-7 miRNA processing by destroying the inhibitory secondary structure in pri-miRNA transcripts (Pandolfini et al., 2019). METTL1 plays an anti-tumor role in colon cancer by activating the let-7e miRNA/HMGA2 axis regulated by m7G (Liu et al., 2020b). METTL1 and its cofactor WDR4 mediated tRNA m7G methylation is necessary for normal mRNA translation and self-renewal and differentiation of embryonic stem cells (Lin et al., 2018). As a new type of RNA methylation, m7G RNA methylation has aroused great research interest (Figure 1E).

## Biological Functions of RNA Methylations

RNAs have the dual identity of information molecules and regulatory molecules. They not only transmit DNA genetic information to proteins, but also regulate many biological processes. The post transcriptional modifications of RNAs lay the foundation for this diverse functions (Shinoda et al., 2020).

### Effects of RNA Methylations on RNA Metabolism

RNA methylations are involved in almost all steps of RNA metabolism, including RNA capping, splicing, translation, nuclear export, and stability. The protein complex DXO/Dom3Z can remove the unmethylated cap, leading to the degradation of the rest of the pre-RNAs (Jiao et al., 2017). M6A is the most common and abundant RNA modification in eukaryotes. The modification of m6A is catalyzed by the METTL3 methyltransferase complex (Du et al., 2018). The RNA demethylases FTO and ALKBH5 can demethylate m6A through an α-ketoglutarate and Fe^2+^ dependent form (Zhang et al., 2016). The METTL3, FTO and ALKBH5 have been shown to play important roles in many biological processes. M6A is enriched in exons of 5ʹ and 3ʹ splicing sites, which is the binding region of serine/arginine-rich protein 2 (SRSF2) regulated by mRNA splicing. Knockdown of FTO results in the increase of m6A level in exon region (Niu et al., 2013). RNA methylations may play important roles in biological processes, from development and metabolism to reproduction.

Nuclear export is an important step in the translation of mRNAs in cytoplasm. RNA methylations, especially the m6A and m5C, play key roles in the process of mRNA transfer from nucleus to cytoplasm (Li and Meng, 2021b). Furthermore, the fragile X intellectual disability protein (FIDP) reads m6A to promote the nuclear export of methylated mRNA targets during neural differentiation. Both the METTL14cKO and Fmr1KO induce nuclear retention of target genes of m6A modified FIDP. The FIDP preferentially binds to m6A modified RNAs, promotes their nuclear export and regulates the neural differentiation through the CRM1. Both m6A and FIDP are essential for the nuclear export of methylation target RNAs in the mechanism of neural differentiation (Edens et al., 2019).

RNA methylations are closely related to mRNA stability, which are involved in the dynamic synthesis and degradation of mRNAs, maintaining the acute amount of mRNAs in different physiological and pathological processes (Kontur and Giraldez, 2017). M6A is the most common modification form of eukaryotic mRNAs. Its readers, such as proteins with YTH domain, recognize it to regulate the stability of mRNAs. IGF2BPs, as a unique family of m6A readers, target thousands of mRNAs by recognizing consistent m6A GGC sequence. The IGF2BPs promote the stability and storage of their target mRNAs (such as MYC) in a m6A dependent manner under normal and stress conditions, and affect subsequent gene expression (Huang et al., 2018). The m6A reader YTHDF2 recognizes the m1A modified sequence in a methylation specific manner. The abundance of m1A modified transcripts increase after the YTHDF2 knockout. YTHDF2, which recognizes the m1A modified RNAs, is associated with transcriptional instability (Seo and Kleiner, 2020).

**FIGURE 2:**
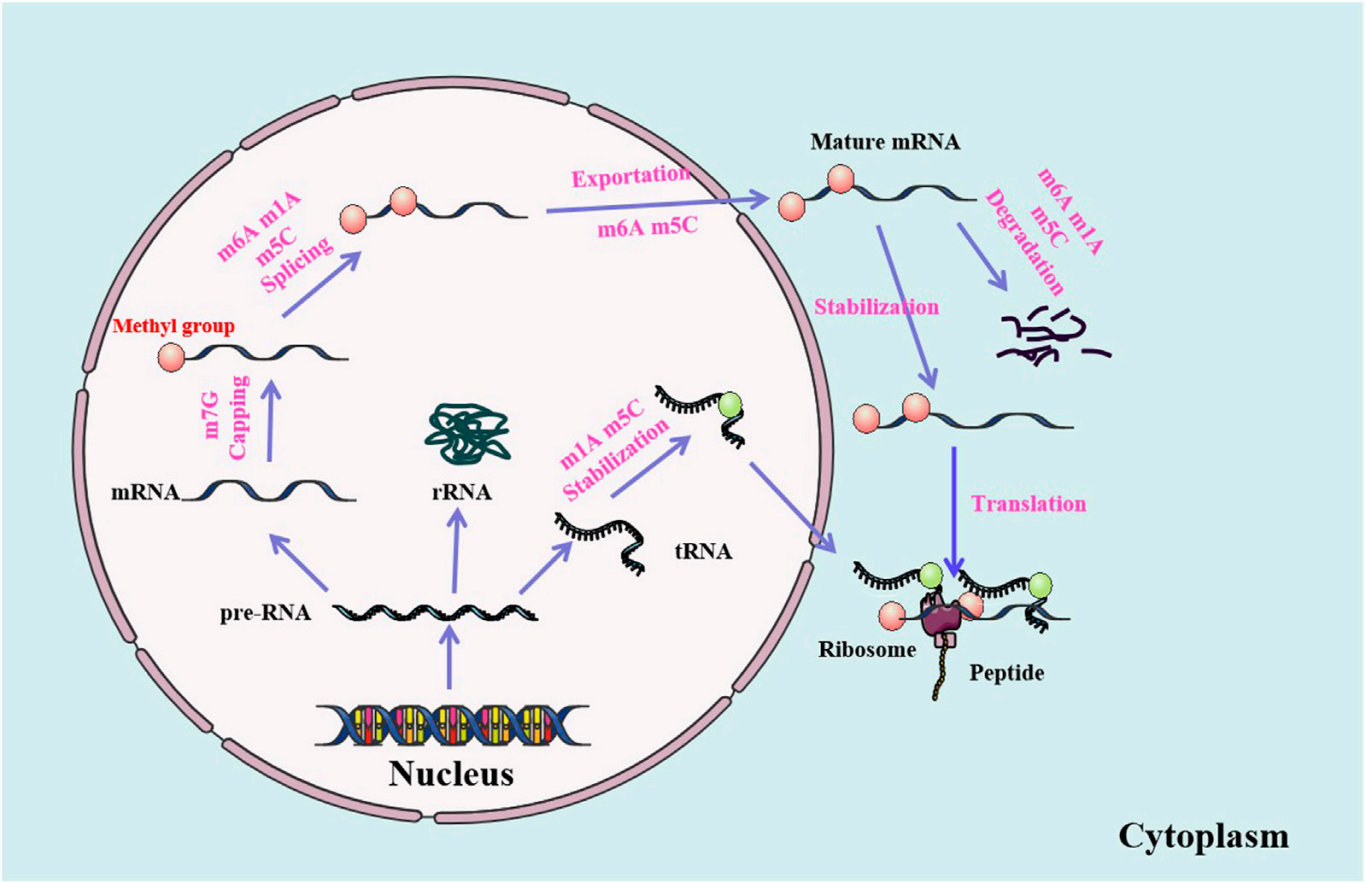
RNA methylations are involved in the modifications and metabolism of RNA molecules. RNA methylations modify the structure of RNAs and participate in the regulation of RNA metabolism, including RNA termination, splicing, stabilization, nuclear export, translation and degradation.

As an m6A eraser, ALKBH5 specifically removes m6A from target mRNAs. The ALKBH5 dependent m6A demethylation controls the splicing and stability of long 3ʹ UTR mRNA in male germ cells. Inactivation of ALKBH5 leads to male sterility in mice (Tang et al., 2018). The FTO of m6A erasers is rich in axons and can be local translated. FTO of axons regulates the modification of axon mRNAs by m6A, which has been proved to be the demethylase mediated mechanisms of controlling the local translation of mRNAs during the development of neurons (Yu et al., 2018) (Figure 2).

### The Roles of RNA Methylations in Biological Functions

RNA methylations are dynamic reversible modifications, which can directly or indirectly affect the biological processes of RNA degradation, translation and splicing, and play important roles in various physiological and pathological processes (Zhang et al., 2019a).

#### RNA Methylations are Involved in the Pathogenesis of Cancers

The disordered regulation of m6A modification has been considered to be closely related to the pathogenesis of liver cancer (Orcutt and Anaya, 2018; Pu et al., 2020). METTL14, the m6A reader, may be involved in the malignant progression of hepatocellular carcinoma by regulating the m6A modification of cysteine sulfinic acid decarboxylase, glutamic-oxaloacetic transaminase 2, and suppressor of cytokine signaling 2 (Li et al., 2020c). The roles of m6A in gastric cancer have made new progress (Wang et al., 2020c). The decrease of m6A is associated with tumor signal transduction and phenotype. High expression of WTAP and FTO indicates a poor prognosis in gastric cancer patients (Guan et al., 2020). Inhibition of m6A (METTL14 knockout) promotes the proliferation and invasion of cancer cells by activating the Wnt and PI3K Akt signals, while elevation of m6A (FTO knockout) reverses these phenotypes (Zhang et al., 2019b).

The RNA methyltransferase NSUN2 is involved in cell proliferation and senescence, and affects the pathogenesis of gastric cancer (Okamoto et al., 2012). It is found that m1A is involved in the mechanism of tumorigenesis and development. For example, in gastrointestinal tumors, the ErbB and mTOR pathways have been identified as regulated by m1A. There is a reliable linkage between m1A and mTOR. Clarification of the imbalance of m1A regulatory factor and its signaling pathway in gastrointestinal tumors is helpful to understand RNA modifications in tumors (Zhao et al., 2019).

Deletion of the RNA demethylase ALKBH5 is associated with the poor clinicopathological and carcinogenesis of pancreatic cancer. Overexpression of the ALKBH5 inhibits the proliferation, migration and invasion of pancreatic cancer cells *in vitro*, while knockout of ALKBH5 gene promotes the progress of pancreatic cancer (Guo et al., 2020a; Tian et al., 2020). Compared with the control group, METTL3 and YTHDF1 are overexpressed in patients with lung adenocarcinoma, and the YTHDF2 is overexpressed in most cases. The METTL3, YTHDF1 and YTHDF2 may be new biomarkers for the prognosis of lung adenocarcinoma (Li et al., 2020a; Zhang et al., 2020d). The regulatory factors of m6A (FTO, IGF2BP3 and RBM15) are closely related to the formation and changes of microenvironment in lung adenocarcinoma (Li et al., 2020b). NSUN2 is the key RNA methyltransferase to add m5C to mRNAs, and its disordered expression is involved in the pathogenesis of non-small cell lung cancer (Van et al., 2019; Chellamuthu and Gray, 2020).

In breast cancer, the expression of METTL3 is decreased, which is closely related to short-term metastasis free survival. The METTL3 knockout can enhance the migration, invasion and adhesion of cancer cells by reducing the level of m6A. The expression of METTL3 was negatively correlated with COL3A1. The METTL3 can inhibit the metastasis of breast cancer cells by increasing the methylation level of m6A and down-regulating the expression of COL3A1 (Shi et al., 2020b). The METTL3 regulates the m6A modification in endometrioid epithelial ovarian cancer independently of METTL14 and WTAP (Ma et al., 2020). Furthermore, The METTL3 promotes the development and progression of thyroid cancer by regulating the m6A methylation on TCF1 (Wang et al., 2020b).

#### The Roles of RNA Methylations in Nervous System

M6A methylation modifying enzymes and binding proteins affect the nervous system from neural stem cells, learning and memory, brain development, axon growth and glioblastoma (Li et al., 2019b). The specific knockout of METTL3 in the mouse nervous system can lead to severe developmental defects in the brain. The reason is that the deletion of METTL3 leads to the deletion of m6A modification, which indirectly leads to the prolongation of RNA half-life and abnormal splicing events (Wang et al., 2018a).

Dynamic RNA methylations play important roles in oligodendrocyte development and myelination in central nervous system (Weng et al., 2018). The deletion of METTL14 in oligodendrocyte cell line results in abnormal splicing of a large number of RNA transcripts. METTL14 knockout also results in decreased number of oligodendrocytes and decreased myelin sheath in the central nervous system, although the number of oligodendrocyte precursor cells (OPC) is normal (Xu et al., 2020). The METTL3-METTL14 complex is an essential regulator for timing of cortical neurogenesis. The deletion of the m6A reading protein YTHDF2 in the embryonic neocortex seriously affects the self-renewal of neural stem/progenitor cell (NSPC) and the spatiotemporal generation of neurons. The YTHDF2 regulates neural development by promoting the degradation of m6A dependent neurodevelopmental related mRNA targets (Li et al., 2018).

Furthermore, m6A regulates the histone methyltransferase EZH2 and affects the neurogenesis and neuronal development (Chen et al., 2019b). N6 methyladenosine RNA modification affects the self-renewal of embryonic neural stem cells through histone modifications (Wang et al., 2018b). Ma et al. (2018) found that the nuclear output of hypermethylated RNAs increased in the cerebellum of ALKBH5 deficient mice exposed to hypobaric hypoxia. The expression of the m6A writers METTL3, METTL14, and WTAP and the erasers ALKBH5 and FTO in mouse cerebellum were spatiotemporal specific. Interestingly, the expression of METTL3 increases and the FTO decreases in AD mice. This suggests that the differentially expressed m6A methylated RNAs may play important roles in AD pathogenesis (Huang et al., 2020a; Han et al., 2020).

#### The Roles of RNA Methylations in Germline Development

RNA modifications play key roles in germ line development. Combined deletion of METTL3, METTL14 and Stra8-GFPCre in advanced germ cells disrupts the spermatogenesis, while single deletion of METTL3 or METTL14 in advanced germ cells shows normal spermatogenesis. The abnormality of METTL3 and METTL14 results in the damage of haploid specific gene translation, which affects sperm production (Neto et al., 2016; Lin et al., 2017). The ALKBH5 is a mammalian RNA demethylase, which can reverse the m6A modification, affects the RNA metabolism and fertility in mice. The demethylation activity of ALKBH5 significantly affects the mRNA export, RNA metabolism, and the assembly of mRNA processing factors in nuclear spots. The expression of m6A mRNA in ALKBH5 deficient male mice increases, and affects the apoptosis of spermatocytes at metaphase of meiosis, resulting in the impairment of fertility (Zheng et al., 2013).

Sperm small non-coding RNAs (sncRNAs) can mediate the intergenerational transmission of paternal acquired phenotypes, including stress and metabolic disorders (Liu et al., 2019b). For example, deletion of mouse tRNA methyltransferase DNMT2 eliminates the transmission of sperm sncRNAs mediated high-fat diet induced metabolic disorders to offspring. DNMT2 deletion prevents the increase of RNA modifications in sperm 30–40 nt RNA fraction induced by high-fat diet, especially m5C modification. The DNMT2 deletion changes the expression profile of sperm small RNAs, indirectly indicating that RNA modifications have a significant impact on the germline development. This is enough to attract people’s attention (Ellis and Stanfield, 2014; Zhang et al., 2018b).

#### RNA Methylation is Involved in Other Physiological and Pathological Mechanisms

In addition to cancers, methylation of m6A on transcripts play key roles in the regulation of downstream molecular events and other biological functions, such as maladjustment of m6A modification related to bacterial resistance, viral replication and pluripotency of embryonic stem cells. In recent years, many studies have found that abnormal methylation of m6A is associated with CVDs, including heart failure, cardiac hypertrophy, aneurysm, vascular calcification and pulmonary hypertension (Wen et al., 2018).

Sepsis is a highly heterogeneous syndrome with different immune states and pathological processes. According to different RNA epigenetics, immune status and biological process, three different subtypes of sepsis have been identified (Salomão et al., 2019). In view of the characteristics of m6A methylation regulatory genes, m6A methylation may be the main reason for the heterogeneity of sepsis (Zhang et al., 2020c).

## Roles of RNA Methylations in CVDs

CVDs are the most common cause of death in developing and developed countries. Although primary prevention has improved, the incidence rate of CVDs has been increasing in recent years (Çakmak and Demir, 2020). Therefore, in-depth study of the pathogenesis of CVDs and looking for new diagnostic and therapeutic biomarkers are particularly urgent. This is of great significance to the early diagnosis and prevention of CVDs (Table 1).

**TABLE 1:** RNA methylation related enzymes in CVDs.

Diseases and cell types	Types of RNA methylation	Enzyme	Expression	Functions	Targets	References
PH samples and hypoxia treated PASMCs	m6A	YTHDF1	Up-regulation	YTHDF1 regulates the PH through translational control of MAGED1	MAGED1	Hu et al. (2021)
Lungs of hypoxia mediated PH model rats	m6A	—	Down-regulation	M6A influences the circRNA-miRNA- mRNA network in hypoxia mediated PH.	CircRNA-miRNA- mRNA network	Su et al. (2020)
PASMCs and hypoxic model rats	m6A	METTL3/YTHDF2	Up-regulation	METTL3/YTHDF2/PTEN axis promotes the hypoxia induced PAH.	PTEN	Qin et al. (2021)
Hypoxia induced PASMCs of PAH mice	m6A	SETD2/METTL14	Up-regulation	SEDT2/METTL14-mediated m6A methylation contributes to the hypoxia induced PAH in mice	RVSP, RV/(LV + S) weight ratio, pulmonary median width	Zhou et al. (2021)
Calcified arteries and indoxyl sulfate induced HASMCs	m6A	METTL14	Up-regulation	METTL14-dependent m6A regulates the vascular calcification induced by indoxyl sulfate	Vascular osteogenic transcripts	Chen et al. (2019a)
Human primary cardiomyocytes, model mice	m6A	METTL3	Up-regulation	METTL3 controls the cardiac homeostasis and hypertrophy	—	Dorn et al. (2019)
Pressure-overloaded mice	m6A	METTL3	Up-regulation	CHAPIR regulates the cardiac hypertrophy by controlling the METTL3 dependent m6A methylation of PARP10 mRNA.	PARP10	Gao et al. (2020)
Clinical human samples, preclinical pig and mouse models, and primary cardiomyocyte cell	m6A	FTO	Down-regulation	FTO dependent m6A regulates the cardiac function during remodeling and repair	Cardiac contractile transcripts	Mathiyalagan et al. (2019)
PBMCs of HFpEF patients	m6A	METTL3, METTL4, KIAA1429, YTHDF2	Up-regulation	M6A regulators in HFpEF uncovers a new transcription-independent mechanism	ERAD pathway, RNA polymerase II, PI3K-Akt signaling pathway	Zhang et al. (2021)
Myocardial cells of heart failure model mice	m6A	FTO	Down-regulation	FTO overexpression inhibits the apoptosis of hypoxia/reoxygenation treated myocardial cells	Mhrt	Shen et al. (2021b)

### RNA Methylations and PH

PH induced by hypoxia is a fatal disease, and there is still a lack of effective treatment (Wang et al., 2021). In human and rodent PH samples and hypoxia treated pulmonary artery smooth muscle cells (PASMCs), the levels of m6A and the YTHDF1 protein are increased. However, YTHDF1 knockout inhibits the PASMC proliferation, phenotype transformation and pH symptoms. It is found that MAGED1 is a direct target of m6A in regulating the pathogenesis of pH, and YTHDF1 recognizes and promotes the translation of MAGED1 in a m6A dependent manner. If the MAGED1 is silenced, the proliferation of PASMCs induced by hypoxia is inhibited by down-regulating the proliferating cell nuclear antigen (PCNA). Interestingly, there is no regulatory effect of YTHDF1 on the MAGED1 expression in METTL3 deficient PASMCs, suggesting that the METTL3 is also involved in the regulation of YTHDF1 on pH pathogenesis (Gerthoffer, 2020; Hu et al., 2021).

However, Xu et al. (2021) found that m6A methyltransferase and demethylase proteins were significantly down-regulated in postnatal hypoxia induced PH in SD rats. The occurrence and persistence of PH may be due to the low expression of METTL3, which affects the levels of PH related genes. The contradictory results of METTL3 expression level may be due to the different tissue samples of model rats or the insufficient number of experimental animals.

CircRNAs play important roles in physiological process, and m6A modification is involved in the regulation mechanisms of circRNAs (Zhang et al., 2020b). It is found that the abundance of m6A in circRNAs decreases significantly under hypoxia *in vitro*. M6A affects the circRNA-miRNA-mRNA network during hypoxia. For example, the levels of circXpo6 and circTmtc3 are down-regulated in PH induced by hypoxia. These two m6A circRNAs, which are related to hypoxia mediated PH, have certain significance in the study of PH pathological mechanisms and treatment (Su et al., 2020).

The METTL3 plays a role in the pathogenesis of pulmonary arterial hypertension (PAH) induced by hypoxia. The study of PASMCs and hypoxic rat models show that the level of METTL3 is up-regulated, and the down-regulation of METTL3 reduces the proliferation and migration of PASMCs. In addition, the m6A binding protein YTHDF2 in PASMCs are up-regulated significantly under hypoxia. YTHDF2 recognizes the PTEN mRNA modified by METTL3 and promotes the degradation of PTEN. The decrease of PTEN further leads to the excessive proliferation of PASMCs by activating the PI3K/Akt signaling pathway. Therefore, the METTL3/YTHDF2/PTEN axis plays an important role in the proliferation of PASMCs induced by hypoxia, which provides a new diagnostic and therapeutic target for the treatment of hypoxic PAH (Liu et al., 2017; Xing et al., 2019; Qin et al., 2021).

The SETD2 catalyzes the trimethylation of lysine 36 on histone 3 (H3K36me3) and is involved in hypoxic PAH (Yao et al., 2020). The expression of SETD2 and m6A writer METTL14 increase in hypoxia induced PASMCs of PAH mice. In the SETD2 specific knockout mouse model, the SMC lacking SETD2 protects mice from hypoxic PAH and significantly reduces related pathological parameters, such as the right ventricular systolic pressure (RVSP), the right ventricular/left ventricular plus septum [RV/(LV + S)] weight ratio, and the pulmonary median width. Furthermore, the deletion of SETD2 reduces the expression of METTL14 and the methylation level of m6A in PAH SMCs. This suggests that the strategy of inhibiting the SETD2/METTL14 may be a feasible method for the treatment of PAH (Zhou et al., 2021).

### RNA Methylations in Hypertension and Atherosclerosis

The roles of m6A methylation in hypertension have not been fully elucidated. However, the high-throughput sequencing show that the methylation of m6A is more abundant in the coding region, 3′UTR and 5′UTR of mRNAs, and the motifs of m6A are relatively conservative. Compared with the control, the mean abundance of m6A in spontaneously hypertensive rat pericytes is decreased. The specific distribution of m6A in the pathogenesis of hypertension suggests that m6A plays an important role in the pathogenesis of hypertension (Guo et al., 2020b; Wu et al., 2019).

METTL14 may be a potential target for clinical treatment of atherosclerosis. METTL14 can enhance the expression of FoxO1 by enhancing its m6A modification, and can also induce endothelial cell inflammation and atherosclerotic plaque formation (Jian et al., 2020). METTL14 mediates the m6A modification of pri-miR-19a, which promotes the processing and maturation of miR-19a, thus promoting the proliferation and invasion of atherosclerotic vascular endothelial cells (ASVEC). METTL14 may be a new target for the treatment of atherosclerosis (Zhang et al., 2020a).

### RNA Methylations and Vascular Calcification

The importance of m6A in various physiological and pathological mechanisms is gradually emerging, and it also plays an important role in vascular calcification (Hou et al., 2020). Chen et al. (2019a) investigated the roles of METTL14 in vascular calcification using clinical human specimens, model rats and HASMCs. It was found that the METTL14 expression increased in calcified arteries and indoxyl sulfate induced HASMCs and the increased METTL14 inhibited the vascular repair function. METTL14 knockout in calcified arteries decreased the level of m6A induced by indoxyl sulfate, resulting in the inhibition of calcification of HASMCs. Furthermore, METTL14 selectively methylated the vascular osteogenic transcripts to promote their degradation, and knockout of METTL14 enhanced the function of vascular repair. The importance of METTL14 mediated m6A in the process of vascular calcification suggests that it may be a new target for the diagnosis and treatment of vascular calcification (Qin et al., 2020).

### RNA Methylations and Cardiac Hypertrophy

The level of m6A in primary cardiomyocytes is up-regulated with the development of cardiac hypertrophy (Liu and Tang, 2019). In the cardiac hypertrophy, genes regulating kinase and intracellular signaling pathway are significantly enriched. Knockout of METTL3 inhibits the progress of cardiac hypertrophy, while overexpression of METTL3 promotes the cardiac hypertrophy. METTL3 mediated m6A modification is enhanced by hypertrophic stimulation, which is necessary for cardiac hypertrophy (Dorn et al., 2019).

PIWI-interacting RNAs (piRNAs) are highly expressed in cardiac hypertrophy, but the underlying mechanism remains unclear. Gao et al. (2020) identified a cardiac hypertrophy related piRNA (CHAPIR). CHAPIR promoted the cardiac hypertrophy and remodeling by targeting the METTL3 mediated methylation of PARP10 mRNA transcripts. For the pressure overload model mice, the absence of CHAPIR significantly reduced the cardiac hypertrophy and restored the cardiac function, while the overexpression of CHAPIR enhanced the pathological hypertrophy response. CHAPIR directly interacted with the METTL3 to block the methylation of PARP10 mRNA transcripts and up-regulated the expression of PARP10. Furthermore, up-regulated PARP10 promoted the single ADP ribosylation of GSK3β and inhibited its kinase activity, eventually leading to the accumulation of nuclear NFATC4 and the progress of pathological cardiac hypertrophy. The CHAPIR-METTL3-PARP10-NFATC4 signal axis may be a new target for the treatment of cardiac hypertrophy (Robson, 2021).

### RNA Methylations and Heart Failure

At present, a large number of mammalian mRNAs are modified by m6A, and the m6A is also an important posttranscriptional regulation mechanism for heart disease discovered in recent years (Wu et al., 2020b). Studies on selective mRNA translation and protein abundance indicate the potential effects of m6A on post transcriptional regulation of cardiac disease related gene expression. M6A may play an important role in cardiac pathophysiology through remodeling of myocardial structure (Hinger et al., 2021).

The imbalance of epigenetic modifications and abnormal gene expression are important mechanisms of heart failure, and the m6A plays an important role in the development of heart failure. For example, model mice with cardiomyocyte knockout of RNA demethylase FTO have impaired cardiac function (Hubacek et al., 2018). About a quarter of the transcripts in human heart tissue show different degrees of m6A modification. In the process of heart failure, the change of m6A modification exceeds the change of gene expression. Among them, the methylation of m6A is mainly related to metabolic and regulatory pathways, and the change of RNA expression is mainly manifested in the change of cardiac structural plasticity. Interestingly, the change of m6A methylation leads to the change of protein abundance, which is independent of mRNA level. This reveals a new transcription independent translation regulation mechanism in the pathogenesis of heart failure (Berulava et al., 2020).

The FTO is closely related to cardiac contractile function. The decreased expression of FTO in the failing mammalian heart and hypoxic cardiomyocytes result in the increased level of m6A in RNAs (Huang et al., 2020b). Increased FTO in the heart failure model mice reduces the ischemia-induced m6A and improves the cardiac contractile function, which may be related to the selective demethylation of cardiac contractile transcripts by FTO. The FTO is also involved in the pathogenesis of myocardial infarction. Overexpression of FTO reduces the myocardial fibrosis and enhances the angiogenesis in mouse model of myocardial infarction. The FTO intervenes the pathogenesis of heart failure through m6A, which provides a new perspective for elucidating the pathogenesis of heart failure (Mathiyalagan et al., 2019).

Heart failure with preserved ejection fraction (HFpEF) is a heterogeneous disease with complex pathogenesis (Simmonds et al., 2020). Compared with health control, the m6A writers METTL3, METTL4 and KIAA1429, the m6A eraser FTO, and the reader YTHDF2 are up-regulated in HFpEF patients. And the level of FTO is also elevated in HFpEF model mice. Gene Ontology (GO) analysis shows that protein folding, ubiquitin dependent ERAD pathway and positive regulation of RNA polymerase II are the three most significant biological processes in HFpEF. KEGG analysis shows that the proteasome, endoplasmic reticulum protein and PI3K-Akt signaling pathway in HFpEF change significantly. The change of expression pattern of m6A methylases in HFpEF reveals a new transcription independent mechanism of gene regulation. It has certain significance for the diagnosis and treatment of HFpEF (Zhang et al., 2021).

**FIGURE 3:**
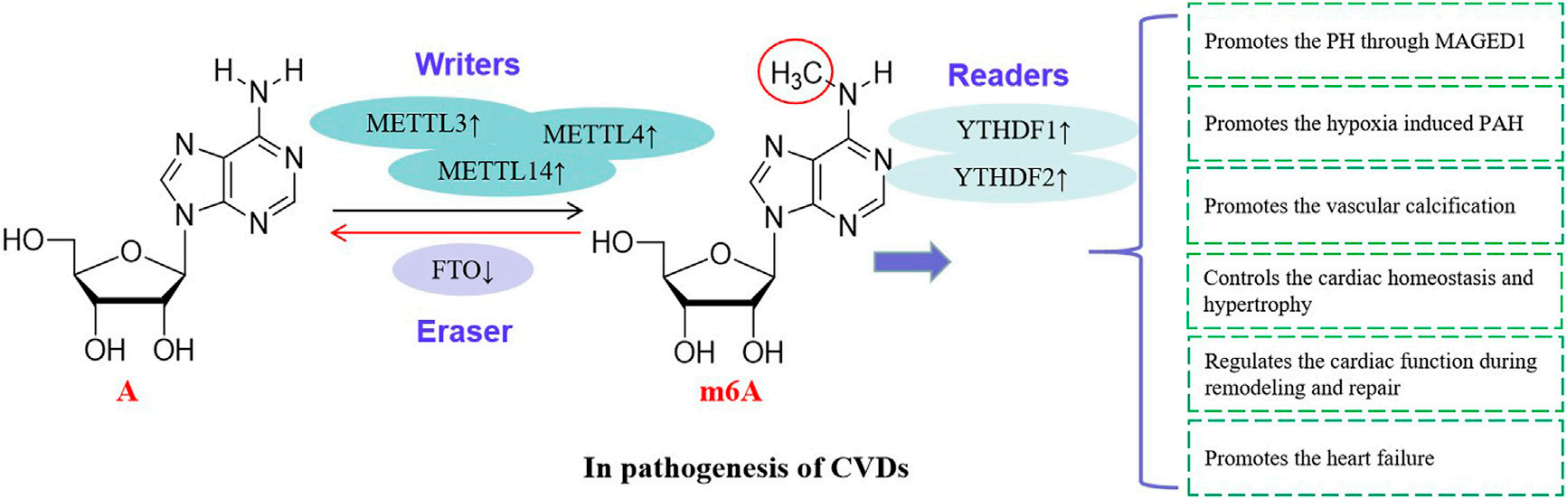
RNA methylations are involved in the CVDs. RNA methylating enzymes catalyze the methylation of m6A and are involved in the pathogenesis of CVDs, suggesting that these enzymes are risk factors of CVDs.

As a competitive endogenous RNA, MALAT1 regulates the hypoxia induced cardiomyocyte proliferation, apoptosis and cell cycle progression by regulating miR-200a-3p/PDCD4 axis (Sun and Zhang, 2019). Heart failure is the end stage of many CVDs, which seriously threatens people’s health. Shen et al. (2021b) established heart failure mice model by transverse aortic constriction or intraperitoneal injection of doxorubicin, and observed the expression of FTO in cardiomyocytes of model mice under hypoxia/reoxygenation condition. In heart failure model mice, the expression of FTO and its target Mhrt were down-regulated. The overexpression of FTO resulted in the decrease of m6A modification of Mhrt and the up-regulation of Mhrt expression, which inhibited the apoptosis of cardiomyocytes induced by the hypoxia/reoxygenation. The result of FTO knockout was just the opposite. These results suggest that FTO overexpression inhibits the cardiomyocyte apoptosis induced by hypoxia/reoxygenation by regulating the m6A modified Mhrt (Song et al., 2019) (Figure 3).

## Conclusion and Future Prospects

In this review, the mechanisms, molecular structures, biological functions of five kinds of RNA methylations (m6A, m5C, m1A, m6Am, m7G) and their effects on CVDs were summarized. RNA methylation “writers” catalyze the installation of m6A on RNAs, and “erasers” remove these modifications. Finally, “readers” of m6A affect the mRNA splicing, export, translation and degradation (Liu and Jia, 2014). In addition, RNA methylations affect the processing of miRNAs and the functions of lncRNAs, and even promote the translation of circRNAs (He et al., 2018). At present, in the field of RNA methylations and CVDs, the disorder of m6A and its impacts on the pathogenesis are gradually revealed, which is worthy of further tracking and attention. M5C, m1A, m6Am and m7G are new types of RNA methylations, and their roles in CVDs need to be observed in future.

The research progress of epigenetics and CVDs always lags behind the research in cancers. The mechanisms of epigenetics in cancer has reference value for the research of CVDs (Dawson and Kouzarides, 2012; Prasher et al., 2020). RNA methylations and related regulatory factors play important roles in the pathogenesis of diseases. Like cancer mechanism research, RNA methylation may be a double-edged sword for CVDs. Some genes promote the development of disease after methylation, while others can promote the development of disease after demethylation. For example, in colorectal carcinoma, SOX2 promotes cancer through methylation catalyzed by METTL3 (Li et al., 2019c), while in breast cancer, BNIP3 promotes the cancer through demethylation catalyzed by FTO (Niu et al., 2019). The same m6A related regulatory factors can play its biological functions through different target genes in the same cancer. For example, in gastric cancer, METTL3 promotes the pathogenesis by catalyzing the methylation of ZMYM1 (Yue et al., 2019) or HDGF (Wang et al., 2020c). In liver cancer, METTL3 and METTL14 play opposite roles in tumor development (Ma et al., 2017; Chen et al., 2018). This difference may be in essence, or it may be due to the insufficient sample size in these studies. Therefore, in the study of CVDs, we should increase the sample size, especially the clinical sample size.

RNA methylations have been confirmed to participate in the development of CVDs (Colpaert and Calore, 2021). RNA methylation related enzymes and their target molecules may become new diagnostic markers and therapeutic targets, thus providing important scientific basis for targeting RNA methylations in the treatment of human CVDs. The study of RNA methylations and CVDs also provides a new perspective for the elucidation of the pathogenesis of these diseases and a new way for clinical treatment. Although the roles of RNA methylations in CVDs have been gradually revealed, there are still many challenges. Firstly, the mechanisms of RNA methylation regulators in CVDs are largely unknown. For example, the role and mechanism of “readers” in the methylation of m6A in CVDs are still unclear. Secondly, although many studies have shown that RNA methylation related regulatory factors and pathways may be new targets in the treatment of CVDs, there is a lack of clinical practice. RNA methylations can affect gene expression in many ways, and its side effects cannot be ignored.

In the future, research on RNA methylations and CVDs should focus on the following aspects. First, a complex regulatory network model of single RNA modification in a single CVD should be constructed. With the deepening of research, gradually improve the network model to find new targets for disease diagnosis and treatment. Second, expand the sample size of clinical cases, repeatedly verify the experimental phenomenon to avoid errors in the experimental results. Thirdly, combined with the *in vivo* research of animal models, develop the RNA methylation targeted therapy to provide new potential options for the treatment of CVDs. Fourth, combined with the exosomes or vesicles secreted by cells, explore the feasibility of RNA modifying factors encapsulated in 100 nm exosomes for therapy of CVDs.

## Abbreviations

CVDs, cardiovascular diseases; AD, Alzheimer’s disease; METTL3, methyltransferase-like 3; METTL14, methyltransferase-like 14; SAM, s-adenosine-l-methionine; WTAP, Wilms tumor 1-associating protein; FTO, fat mass and obesity-associated protein; hm6A, N(6)-hydroxymethyladenosine; PH, pulmonary hypertension; PAH, pulmonary arterial hypertension; PCNA, proliferating cell nuclear antigen; RVSP, right ventricular systolic pressure; piRNAs, PIWI-interacting RNAs; CHAPIR, cardiac hypertrophy related piRNA; HFpEF, heart failure with preserved ejection fraction; GO, gene ontology; YTHDF, YTH-domain-containing family proteins; IGF2BP, insulin-like growth factor 2 mRNA-binding proteins; eRNAs, enhancer RNAs; NSUN, NOL1/NOP2/SUN domain; RRM, RNA recognition motif; YBX1, Y-box binding protein 1; IGF1R, insulin-like growth factor 1 receptor; Am, 2′-O-methyladenosine; SRSF2, serine/arginine-rich protein 2; FIDP, fragile X intellectual disability protein; HNSCC, neck square cell carcinoma; OPC, oligodendrocyte precursor cells; NSPC, neural stem/progenitor cell; sncRNAs, small non-coding RNAs; SMCs, smooth muscle cells; GBM, glioblastoma; ASVEC, atherosclerotic vascular endothelial cells; ALKBH5, alkylation repair homologous protein 5; DAS28, disease activity score 28; C3, complement 3; LMR, lymphocyte to monocyte ratio; PBMCs, peripheral blood mononuclear cells; NLR, neutrophil lymphocyte ratio; Ts, T suppressor; Treg, regulatory T; Foxp3, forkhead box P3.
